# Atmospheric Pressure Room Temperature Plasma Jets Facilitate Oxidative and Nitrative Stress and Lead to Endoplasmic Reticulum Stress Dependent Apoptosis in HepG2 Cells

**DOI:** 10.1371/journal.pone.0073665

**Published:** 2013-08-27

**Authors:** Shasha Zhao, Zilan Xiong, Xiang Mao, Dandan Meng, Qian Lei, Yin Li, Pengyi Deng, Mingjie Chen, Min Tu, Xinpei Lu, Guangxiao Yang, Guangyuan He

**Affiliations:** 1 The Genetic Engineering International Cooperation Base of Chinese Ministry of Science and Technology, The Key Laboratory of Molecular Biophysics of Chinese Ministry of Education, College of Life Science and Technology, Huazhong University of Science & Technology (HUST), Wuhan, China; 2 State Key Laboratory of Advanced Electromagnetic Engineering and Technology, School of Electrical and Electronic Engineering, Huazhong University of Science & Technology (HUST), Wuhan, China; University Paul Sabatier, France

## Abstract

Atmospheric pressure room temperature plasma jets (APRTP-Js) that can emit a mixture of different active species have recently found entry in various medical applications. Apoptosis is a key event in APRTP-Js-induced cellular toxicity, but the exact biological mechanisms underlying remain elusive. Here, we explored the role of reactive oxygen species (ROS) and reactive nitrogen species (RNS) in APRTP-Js-induced apoptosis using *in vitro* model of HepG2 cells. We found that APRTP-Js facilitated the accumulation of ROS and RNS in cells, which resulted in the compromised cellular antioxidant defense system, as evidenced by the inactivation of cellular antioxidants including glutathione (GSH), superoxide dismutase (SOD) and catalase. Nitrotyrosine and protein carbonyl content analysis indicated that APRTP-Js treatment caused nitrative and oxidative injury of cells. Meanwhile, intracellular calcium homeostasis was disturbed along with the alteration in the expressions of GRP78, CHOP and pro-caspase12. These effects accumulated and eventually culminated into the cellular dysfunction and endoplasmic reticulum stress (ER stress)-mediated apoptosis. The apoptosis could be markedly attenuated by N-acetylcysteine (NAC, a free radical scavenger), which confirmed the involvement of oxidative and nitrative stress in the process leading to HepG2 cell apoptosis by APRTP-Js treatment.

## Introduction

Unlike the plasma in the medical sense, physical plasmas are regarded as the fourth state of matter and consist of free electrons, various ions, atoms and most importantly, the free radicals. This makes physical plasmas the unique properties compared to solids, liquids or gases. Recently, atmospheric pressure room temperature plasma jets (APRTP-Js) have been proved to have potential applications in blood coagulation [1,2], tissue sterilization [1], cancer therapy [3–5], root canal treatment [6,7], wound care [8] and diverse other applications [9–14]. The advantages of APRTP-Js include their dry procedure, high reactive efficiency, no hazardous residual, friendly to temperature sensitive material, easy operation, and so on. APRTP-Js emit a mixture of different biological active species such as reactive nitrogen species (RNS) like nitric oxide (NO) and reactive oxygen species (ROS) like superoxide anion (O_2_^·-^), hydroxyl radicals (OH^·^), ozone (O_3_) and singlet oxygen ( ^1^O_2_) mainly [15,16].

Both ROS and RNS are double-edged swords that can interact with living cells to regulate cellular functions ranging from cell proliferation to cell death [17]. At low concentrations, these radical species can act as signaling molecules to modulate the proliferation, differentiation and other actions of cells [18,19]. However, at high concentrations, they may result in oxidative and/or nitrative stress and damage to cellular constituents, including nucleic acids, membrane lipids, and proteins which can influence various physiological and pathological processes involving metabolism, inflammation, cell signaling, immunity, transcriptional regulation, and apoptosis [20,21]. To maintain the ROS/RNS in check to prevent increase in oxidative/nitrative stress, mammalian cells have developed a sophisticated defense system to eliminate the endogenous and exogenous free radicals [22–24]. The intracellular defense system is composed of nonenzymatic antioxidants such as glutathione and antioxidant enzymes such as superoxide dismutase (SOD), catalase, glutathione peroxidase (GPx) and glutathione reductase (GR). These antioxidants work in tandem to eliminate free radicals. The SOD family, a metal-containing enzyme that exists in the cytoplasm (Cu/Zn–SOD) or mitochondria (MnSOD), catalyzes the dismutation of superoxide anion (O_2_^·-^) to hydrogen peroxide (H_2_O_2_). Subsequently, toxic H_2_O_2_ is decomposed into non-toxic water (H_2_O) and oxygen (O_2_) by catalase or GPx. GPx catalyzes the deoxygenation of H_2_O_2_ in the presence of reduced glutathione (GSH) to form H_2_O and oxidized glutathione (GSSG). The reaction of GPx is complemented *via* GR by converting GSSG to GSH [25]. An appropriate balance between the free radicals and scavenging antioxidants is important for cellular resistance to nitrative and oxidative stress. However, this balance can be destroyed by various factors, either intrinsic or extrinsic. When the generation of ROS/RNS exceeds the antioxidant capacity of cells, the free radicals can’t be effectively scavenged, causing oxidative/nitrative damage in cells, thus apoptosis may happen.

Tyrosine nitration is a post-translational modification of proteins that commonly occurs when cells respond to oxidative and nitrative stress. Overproduction of RNS/ROS and/or overwhelmed antioxidant systems are responsible for it [26]. Nitrotyrosine is considered to be a biomarker of RNS-dependent oxidative stress. This nitrative modification is characterized by selectively modifying the tyrosine residues exposed to intermolecular acidic or basic environment through a series of oxidative processes mediated by RNS [27]. Meanwhile, the occurrence of oxidative stress in cells is often accompanied with the formation of protein carbonyl groups [28]. These oxidative and nitrative modifications of proteins may lead to structural and functional alterations, as well as the changes in the rate of proteolytic degradation that reduce cells to dysfunction [29–31]. Therefore, elevated levels of protein carbonyl groups and nitrotyrosine are often used as indicators of oxidative and nitrative damage, which may be directly involved in the onset and/or progression of apoptosis in cells.

Moreover, the redox imbalance in cells could directly and/or indirectly affect the endoplasmic reticulum (ER) homeostasis, resulting in ER stress in cells [32,33]. The functions of ER involve the maintenance of intracellular calcium homeostasis, synthesis of lipid and proteins, as well as their sorting and trafficking. The primary purpose of the ER stress is to relieve the stressful perturbance to maintain a fine ER homeostasis. However, it will trigger apoptosis for intense and persistent ER stress [34,35]. ER stress-mediated apoptosis is one of the main pathways of apoptosis. Many proteins are involved in this process. Glucose-regulated protein 78 (GRP78) is an ER chaperone protein that plays an important role in protein folding, assembly, trafficking, as well as the maintenance of intracellular calcium homeostasis [36]. In the case of ER stress, the expression of GRP78 increases markedly. They may associate with the mutant or defective proteins that are incorrectly folded, thus preventing their export from the ER lumen, to maintain the homeostasis of cells. C/EBP-homologous protein (CHOP) is a member of the C/EBP family of transcription factors. Under physiological conditions, the expression of CHOP is very low, but it will be significantly up-regulated in response to ER stress [37]. Caspase12 is a protein located to ER and can be specifically activated by stimuli that elicit ER stress [38]. CHOP and caspase12 are critical executioners involved in the ER stress-mediated apoptosis.

It is well established that APRTP-Js can emit numerous ROS and RNS and apoptotic cell death is a key event in plasma-induced cytotoxicity. Previous studies have focused mainly on the capability of plasma to induce cell apoptosis, however, the exact biological mechanism during APRTP-Js treatment remains elusive, and optimal therapeutic approaches for biomedical applications remain undefined. The aim of this study was to investigate the role of ROS/RNS, whether plasma exposure can cause oxidative/nitrative damage and if so, to delineate the underlying biological mechanism by which HepG2 apoptosis is initiated following plasma exposure *in vitro*. A better understanding of this process may provide us new insights into the evaluation of plasma medicine with the safe utilization of plasma technology.

## Materials and Methods

### Cell Culture

Human hepatoma cancer cell lines (HepG2) were purchased from China Center for Type Culture Collection (CCTCC, Wuhan, China). The cells were cultured in high-glucose Dulbecco’s modified Eagle’s medium (DMEM, HyClone, Logan, UT) supplemented with 10% (v/v) heat-inactivated fetal bovine serum (FBS, HyClone) in an incubator containing a humidified atmosphere of 5% CO_2_ at 37 ^◦^C. After attaining confluence, the cells were detached with 0.25% trypsin (HyClone) and seeded onto 6-well plates (Corning, New York, USA) at the density of 2×10^5^ cells each well. Prior to APRTP-Js treatment, culture medium was changed to 800 µL of phosphate-buffered saline (PBS). Direct APRTP-Js treatment on cells was carried out in open air, 10 mm upper the adherent cells of each well. After treatment for the indicated times, PBS was removed and cells were continuously cultured in fresh medium for 24 h (unless stated otherwise) to perform further analysis. Sham-treated controls were kept in PBS at room temperature during the experimental procedure to ensure equal treatment conditions.

N-acetylcysteine (NAC, Beyotime, Jiangsu, China) was used as an antioxidant dissolved in PBS at a concentration of 10 mM to scavenge the free radicals.

### Optical Emission Spectroscopy and *In Vitro* APRTP-Js Treatment

**Figure 1:**
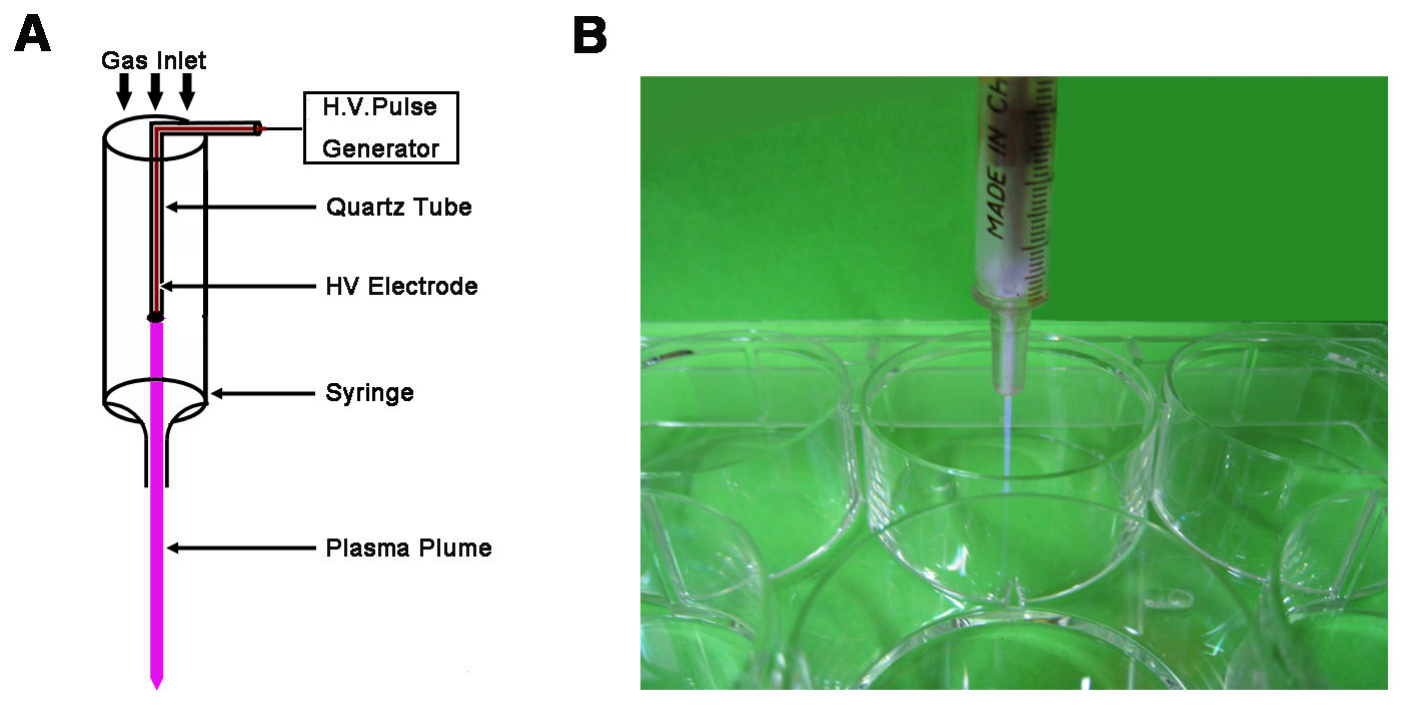
Schematic of APRTP-Js used in the HepG2 cell treatment. (A) Schematic of the APRTP-Js device and (B) photograph of APRTP-Js acting on the HepG2 cells.

A single electrode atmospheric pressure room temperature plasma jet device was used to treat the HepG2 cells as shown in Figure 1, in which (A) was the schematic of the device and (B) was the photograph of APRTP-Js acting on the HepG2 cells. A copper wire was inserted into a 4 cm long quartz tube as the high-voltage (HV). This HV electrode was then inserted into a hollow barrel of a syringe to form the main body of the plasma jet device. Detailed information about the device could be found in Ref. [39]. For all the experiment in this paper, an applied voltage of 8 kV, a frequency of 8 kHz, and pulse width of 1600 ns were fixed. The working gasses He/O2 (1%) with flow rate of 1 L/min were used. Optical emission spectroscopy was carried out on a half meter spectrometer (Princeton instruments, Acton SpectraHub 2500i, USA) to record the spectral line of excited state species in plasma.

For *in vitro* APRTP-Js treatment, distance between the nozzle of the plasma jet device and the samples was ~10 mm. Samples were divided into 4 groups with APRTP-Js treatment of 0 s (sham-treated control), 240 s (4 min), 480 s (8 min) and 720 s (12 min), respectively. In order to make the experiments more accurate, we used “second” as the time unit in our study. Triplicate samples of each group were carried out consecutively at each time point to establish statistical validity.

### Apoptosis Assay

To confirm the pro-apoptotic effect of plasma, cell apoptosis was measured by flow cytometry using an annexin V-FITC apoptosis detection kit (BioVision, Palo Alto, CA). Briefly, nonadherent and adherent cells were collected. Then cells were suspended in 500 µL binding buffer and stained with annexin V-FITC and propidium iodide (PI) at room temperature for 5 min in the dark. After removing the unbound annexin V-FITC and PI by centrifugation, the cells were resuspended in excess binding buffer. For each measurement, at least 10,000 cells were analyzed by FACScan flow cytometer (Beckman Coulter, USA).

To analyze the typical morphological signs of apoptotic nuclei, an additional Hoechst 33342 staining was performed. 24 h after APRTP-Js treatment, cells were incubated with Hoechst 33342 (Beyotime, Jiangsu, China) at room temperature for 5 min. Hoechst 33342-stained cells were analyzed and photographed under a Nikon fluorescent microscope (80i, Nikon, Tokyo, Japan). Cells were scored as apoptotic when nuclei were stained brightly or condensed with Hoechst 33342 and healthy when stained dimly.

### Measurement of ROS and RNS Generation

To monitor the ROS generation, cells were harvested, washed with serum-free DMEM culture medium and incubated at 37°C for 20 min with 10 µM DCFH-DA (Beyotime, Jiangsu, China) which could be oxidized to fluorescent DCF by ROS within the cell. Levels of ROS were determined as the fluorescence of DCF by flow cytometry. Tracings were obtained by displaying the log fluorescence of the samples generated against the background staining of cells.

For RNS detection, the level of NO was determined using Griess-assay kit (Beyotime, Jiangsu, China) according to the manufacturer’s protocol. In brief, the cells were washed twice with ice-cold PBS, followed by being lysed using cell lysis buffer (Beyotime, Jiangsu, China). The lysates were collected and centrifuged at 4°C for 5 min at 12,000g. Assays were performed using the supernatants, and a standard curve using NaNO_2_ was generated for quantification. Briefly, 50 µL of the supernatants or standard NaNO_2_, as well as 50 µL of Griess reagent I and 50 µL of Griess reagent II were added into a 96-well plate. After reacting for 10 minutes at room temperature, the absorbance was measured at 560 nm on a microplate reader (Sunrise, Tecan). NO concentrations were calculated with a reference to the standard curve of NaNO_2_ generated by known concentrations.

### Assay of Nitric Oxide Synthase (NOS) Activities

NOS enzymatic activities of cells by APRTP-Js treatment were determined using a NOS assay kit following the manufacturer’s instructions (Nanjing Jiancheng Bioengineering Institute, Nanjing, China). The assay for NOS activities depends on the rate of conversion of arginine (Arg) to NO, which can further react with nucleophilic substances to produce chromophoric compound that has a peak absorbance at 530 nm. iNOS (inducible NOS) activities can be determined based on the fact that cNOS [constitutive NOS including neuronal NOS (nNOS) and endothelial NOS (eNOS)] are Ca^2+^ -dependent, while iNOS is Ca^2+^ -independent [40]. One NOS enzymatic unit was defined as the capacity of forming 1 nmol NO in 1 min per milligram of protein.

### Antioxidant Capacity Detection of Cells

The antioxidant capacity of HepG2 cells was evaluated 24 h after APRTP-Js treatment. The cells were collected and lysed using cell lysis buffer. The samples were centrifuged and the supernatants were used for antioxidant activities assay.

Determination of GSH concentration: The GSH concentration was evaluated using a commercially available GSH/GSSG assay kit (Beyotime, Jiangsu, China) according to the DTNB-GSSG recycling method. DTNB [5, 5’-dithio-bis-(2-nitrobenzoic acid)], known as Ellman’s reagent, is developed for the detection of thiol compounds. Since DTNB can react with GSH to generate TNB (2-nitro-5-thiobenzoic acid) which is a yellow colored product that can be measured at 405 nm, GSH concentration can be determined. Assay was performed using supernatants following the manufacturer’s instructions. The absorbance values were monitored at 405 nm on the microplate reader.

Determination of SOD activity: SOD activity in lysates was quantified using a SOD analysis Kit-WST (Beyotime, Jiangsu, China) by a competitive assay. Briefly, 20 µL lysates were incubated with assay reagent containing xanthine, xanthine oxidase, and a water-soluble tetrazolium salt, WST-1. The superoxide radicals generated from the xanthine substrate by xanthine oxidase reduce WST-1 to WST-1 formazan, which absorbed maximally at 450 nm. The WST-1 reduction could be inhibited by SOD as it catalyzed the dismutation of superoxide ions to O_2_ and H_2_O_2_. The reduction of WST-1 was measured spectrophotometrically. One unit of SOD activity was defined as the amount of protein that inhibited the WST-1 reduction to 50% of maximum. Enzymatic activity was expressed in units per milligram of protein.

Determination of catalase activity: Catalase activity was detected using a catalase assay kit (Beyotime, Jiangsu, China). Briefly, samples were treated with sufficient H_2_O_2_ for decomposition by catalase for an exact time, and the remaining H_2_O_2_ coupled with a substrate was treated with peroxidase to generate a red product, which absorbed maximally at 520 nm. Catalase activity was thus determined by measuring the decomposition of H_2_O_2_ spectrophotometrically. The enzymatic activity was calculated by comparison with the standard and was expressed in units per milligram of protein.

To calculate each enzymatic activity, the protein concentrations were measured using a BCA protein assay kit (Beyotime, Jiangsu, China).

### Assay of Oxidative Damage

To assess the oxidative damage of cells, the overall formation of protein carbonyl content was determined using a protein carbonyl assay kit (Cayman Chemical, Ann Arbor, USA). Briefly, cell lysates were derivatized with DNPH which could react with carbonyls to form the corresponding hydrazone that had a maximum absorbance of 370 nm. Samples were incubated in the dark for 1 h at room temperature and vortexed every 15 min. Because of the methodology of the assay, the protein carbonyl assay would react with carbonyls from both proteins and lipids. To discriminate between the two, the proteins were precipitated with 20% trichloro acetic acid (TCA). Then, a mixture of ethanol and ethyl acetate (1:1) was utilized to get rid of the contaminants such as lipids, underivatized proteins and free DNPH. So the signal from the assay would only come from protein carbonyls, not from lipid carbonyls. The protein carbonyl content was expressed as nmol carbonyl per milligram of protein.

### Western Blot Analysis for Protein Nitration and Expression

Protein extraction was performed as soon as HepG2 cells were cultured for the indicated time periods. For Western blot analysis, 30 µg of proteins per lane were separated on 10% sodium dodecyl sulfate-polyacrylamide gel electrophoresis (SDS-PAGE) and transferred onto nitrocellulose membrane (Millipore, MA, USA). After being blocked with 5% nonfat dried milk, the membrane was incubated with the respective primary antibodies specific for nitrotyrosine (Santa Cruz Biotechnology, USA), GRP78 (Santa Cruz), CHOP (Santa Cruz), pro-caspase12 (Boster, Wuhan, China), iNOS (Boster) and β-actin (Boster). Subsequently, HRP-conjugated secondary antibodies of goat anti-mouse or goat anti-rabbit (Pierce Biotechnology, Rockford, IL) were used to detect the corresponding primary antibodies. Immunoblot was developed using enhanced chemiluminescence (Millipore, USA) on ChemiDoc XRS+ (Bio-Rad, USA).

### Measurement of Intracellular Free calcium

To monitor the effect of APRTP-Js treatment on intracellular calcium (Ca^2+^) homeostasis, intracellular free Ca^2+^ was measured using calcium probe Fluo-3/AM which could permeate into cells where it was cut into Fluo-3. Fluo-3 resorted within cells and combined with intracellular Ca^2+^ to form a fluorescent compound, whose fluorescence could be detected at excitation wavelength of 488 nm and emission wavelength of 525 nm. Briefly, after APRTP-Js treatment, HepG2 cells were harvested and then loaded with the 5 µM Fluo-3/AM (Beyotime, Jiangsu, China) for 45 min at 37°C. Excess Fluo-3/AM was removed by washing three times with PBS, and additional 30 min was allowed to fully hydrolyze Fluo-3/AM. Free Ca^2+^ levels in individual cells were measured using a FACScan flow cytometer.

### Statistical Analysis

GraphPad Prism 5.0 software was used for statistical analysis. Results were expressed as means + SEM of triplicate samples and reproducibility was confirmed in at least three independent experiments. Student’s t-tests were used to compare the two groups. Differences were considered significant at P<0.05 or P<0.01.

## Results

### APRTP-Js Emitted ROS and RNS

**Figure 2:**
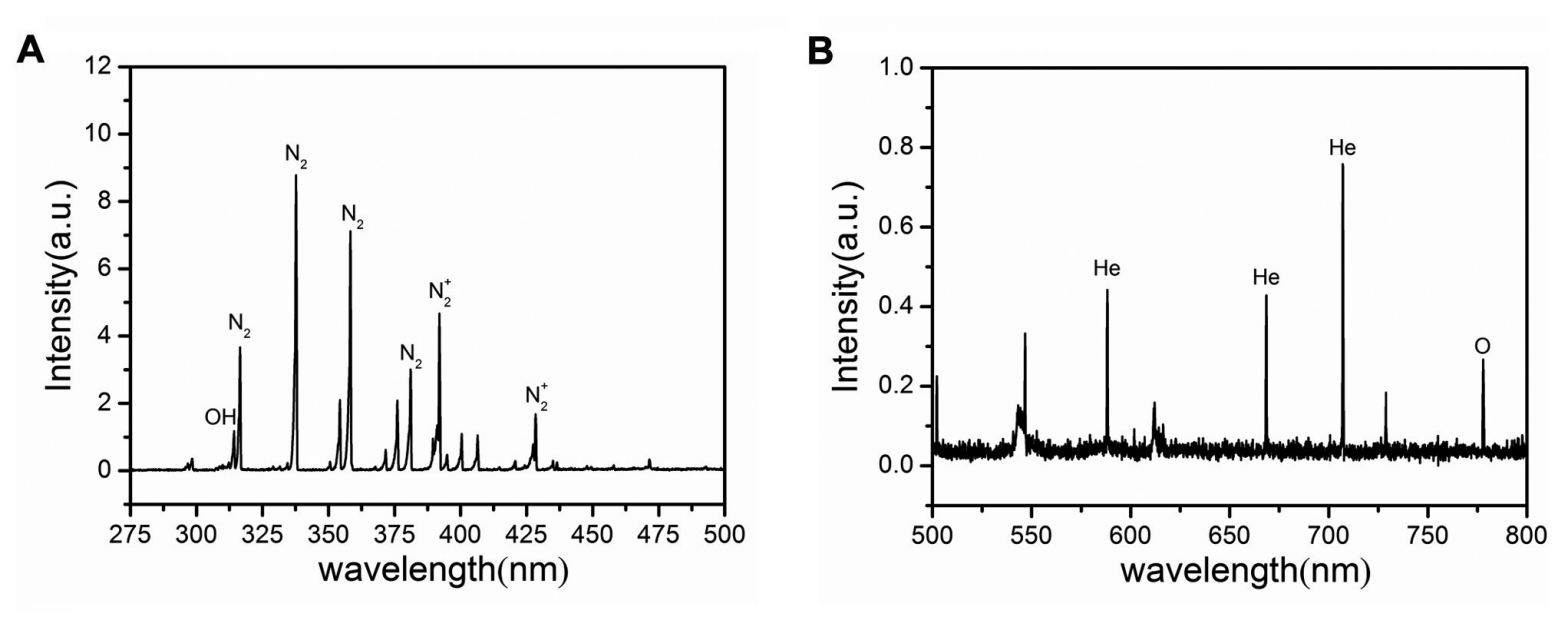
Typical emission spectra of the plasma emitted by APRTP-Js. (A) 275-500 nm (B) 500-800 nm.

Optical emission spectroscopy was carried out to record the spectral line of excited state species in plasma. Figure 2 is the typical emission spectra (275-800nm) of the plasma emitted by the APRTP-Js. Large number of excited OH^·^, N_2_^+^, He and O were observed mainly. Because of the limitations of this method, we could just detect the excited state of the species. These excited species could interact as soon as being emitted, forming NO, O_2_^·-^ and other active species.

### APRTP-Js Treatment Induced Apoptosis in HepG2 Cells

To confirm the pro-apoptotic effect of plasma, annexin V-FITC/PI and Hoechst 33342 staining were utilized in HepG2 cells treated with APRTP-Js.

**Figure 3:**
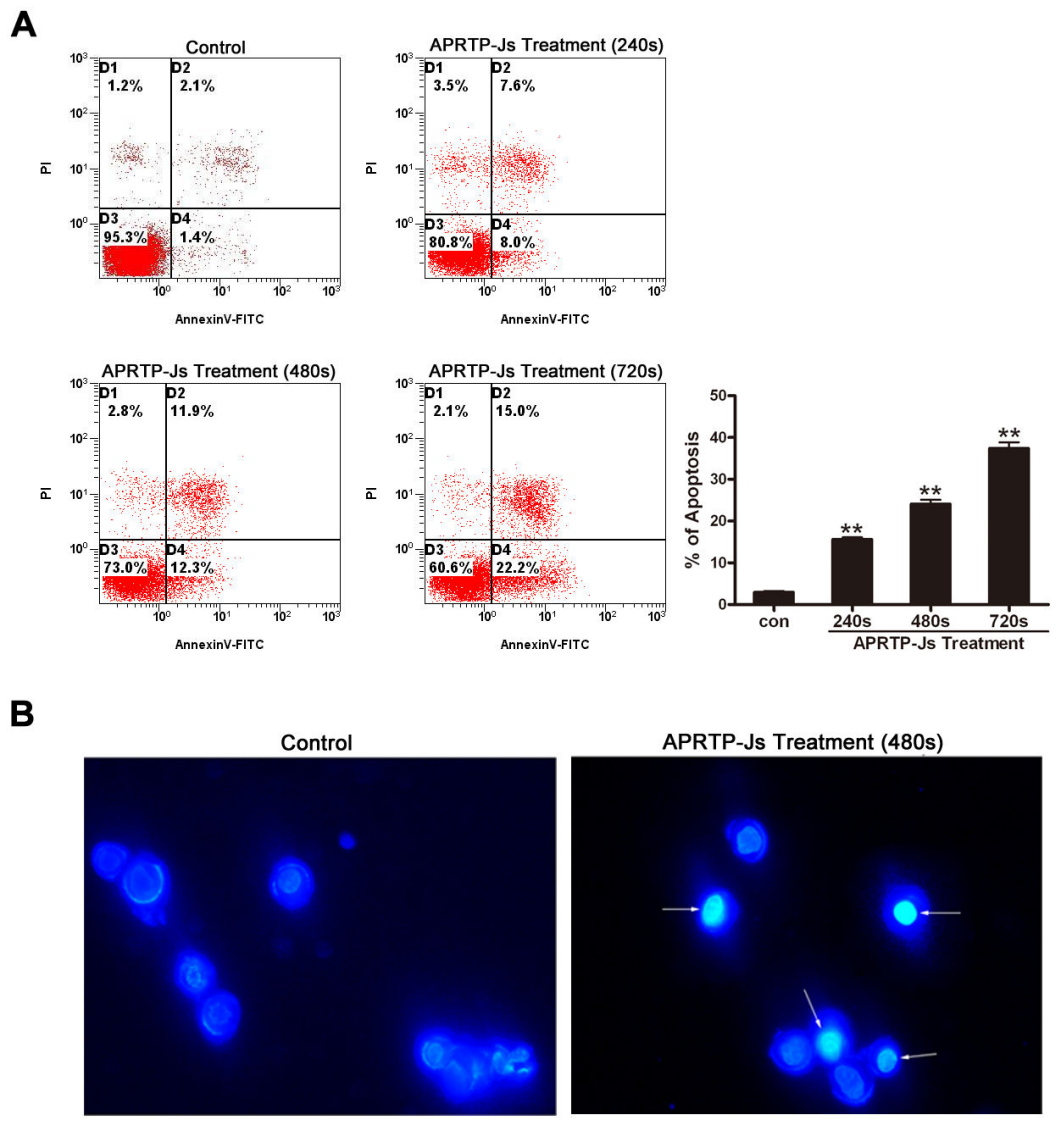
APRTP-Js treatment induced apoptosis in HepG2 cells. Cells were treated by APRTP-Js for 240 s, 480 s and 720 s, and then cultured for 24 h. (A) Cells were double stained with annexin V-FITC and PI and analyzed by flow cytometry. Cells that stained positive for annexin V-FITC and negative for PI were undergoing early stage of apoptosis; Cells that stained positive for both annexin V-FITC and PI were in the end stage of apoptosis; Cells that stained negative for both annexin V-FITC and PI were alive and not undergoing measurable apoptosis. Percentage of apoptotic cells (annexin V-FITC positive) was shown by histogram. (B) Observation of Hoechst 33342 apoptosis staining by ﬂuorescence microscopy. Examples of typical apoptotic nuclei were indicated by white arrows.

Annexin-V is a phospholipid binding protein with high affinity for phosphatidylserine (PS), which is an important component of the cell membrane. Molecules of PS normally localize to the inner surface of the cell membrane, while in apoptotic cells, they are translocated to the outer surface of membrane where annexin-V can easily bind with them. Using this method, the apoptotic cells can be easily distinguished. The HepG2 cells were treated with APRTP-Js at three different doses, viz, 240 s, 480 s and 720 s. Sham-treated cells were used as control. As shown in Figure 3A, 24 h after APRTP-Js treatment, the number of total apoptotic cells in control group was ~3.5%, compared with ~15.6%, 24.2% and 37.2% respectively in APRTP-Js-treated groups, indicating that the plasma could obviously induce apoptosis in HepG2 cells in a dose-dependent manner as seen by the increase in annexin-V positive cells.

Cells undergoing apoptosis usually develop characteristic morphological changes, including chromatin condensation and degradation of DNA into nucleosomal fragments. Thus, we also observed morphological changes of nuclei. Hoechst 33342 staining showed that chromatin condensation and nuclear fragmentation occurred in cells with 480 s of APRTP-Js treatment; while in control group, round and large nuclei appeared with regular contours, and cells with condensed chromatin were rarely seen in Figure 3B.

### APRTP-Js Treatment Facilitated ROS and RNS Accumulation in HepG2 cells

APRTP-Js can produce various ROS and RNS, which are important prooxidant that can induce cell apoptosis. To investigate the roles of these species in plasma-induced apoptosis, intracellular levels of ROS and RNS were detected.

**Figure 4:**
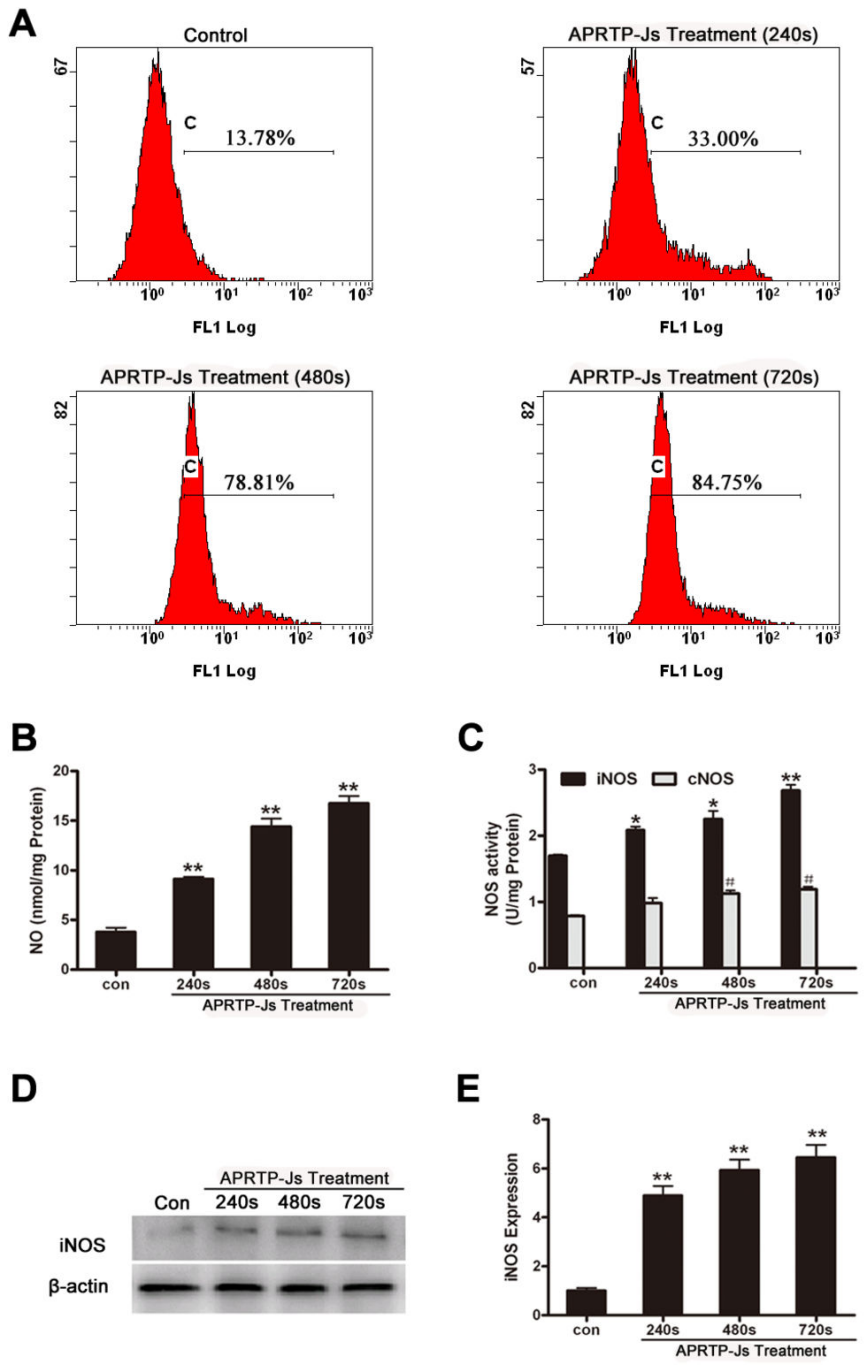
APRTP-Js treatment facilitated ROS and RNS accumulation in HepG2 cells. (A) Measurement of ROS generation in HepG2 cells 24 h after APRTP-Js treatment. Cells were stained with DCFH-DA probe and DCF fluorescence was measured using a flow cytometer. (B) Effect of the APRTP-Js treatment on the intracellular NO concentration in HepG2 cells after 24 h culture. (C) The influence of APRTP-Js treatment on NOS enzymatic activities in HepG2 cells after 24 h culture. The statistical significance was carried out in comparison with the corresponding control value (*P < 0.05 and **P < 0.01 versus iNOS control, **^#^** P < 0.05 versus cNOS control). (D) Effect of plasma on the expression of iNOS. Detection of β-actin was carried out to confirm the equal loading of proteins. (E) The corresponding densitometric analysis of iNOS. Data were normalized using the β-actin signal. Values are means ± SEM obtained from three independent experiments. The asterisk represents statistical significance in comparison with control (*p < 0.05 and **p < 0.01).

Initially, the HepG2 cells were loaded with DCFH-DA to evaluate the accumulation of intracellular ROS after APRTP-Js treatment by flow cytometry. In Figure 4A, the horizontal axis represented the fluorescence intensity, and the vertical axis was the number of cells. The percentage of DCF-positive cells in groups treated with 240 s, 480 s and 720 s of plasma was 33.00%, 78.81% and 84.75%, respectively. The levels were obviously higher than those of control (13.78%), indicating the significant accumulation of intracellular ROS.

Furthermore, the intracellular RNS was evaluated by monitoring the NO production. As shown in Figure 4B, when the APRTP-Js treatment time was varied from 240 s to 720 s, the NO concentrations increased significantly by ~2, ~3.5 and ~4.2 folds compared to control, indicating the increased production of intracellular NO species in cells with APRTP-Js treatment.

The accumulation of ROS and RNS in HepG2 cells by APRTP-Js treatment were consistent with the results reported by Yan et al. [16]. It’s well established that APRTP-Js can produce ROS and RNS, which are short-lived reactive species. However, the ROS and RNS production showed above was detected 24 h after APRTP-Js treatment. The prolonged high levels of ROS and RNS indicated that APRTP-Js treatment caused long-term physiological and biochemical changes within the cells. Generation of intracellular ROS is an inevitable outcome of oxygen-dependent respiration within the cells especially suffering damage. So, where does the RNS come from? Is there any other ways to produce intracellular RNS besides the RNS emitted by plasma which can diffuse into the cells to form nitrite with high stability? And, how it works to maintain the long-term induction of apoptosis by plasma?

### APRTP-Js Treatment Regulated the Expression and Activities of NOS in HepG2 Cells

Because the increased production of intracellular NO species is always associated with the up-regulation of iNOS expression or the activation of NOS enzymes, the NOS activities and expression were examined. As shown in Figure 4C, both of the total cellular iNOS and cNOS enzymatic activities were increased with plasma-dose dependency. The iNOS enzymatic activity was increased to ~1.5 folds in maximum with APRTP-Js treatment for 720 s, compared to control. However, cNOS activity was just increased slightly. Meanwhile, the expression of iNOS was up-regulated (Figure 4D and E). Because cNOS is Ca^2+^ -dependent and iNOS is Ca^2+^ -independent, the increase of cNOS activity may be related to the regulation of intracellular calcium while the raise in iNOS enzymatic activity should be attributed to the up-regulation of its expression.

The noteworthy observation of this study was that the increase of iNOS and cNOS activities did not parallel with the NO production. Although APRTP-Js treatment for 720 s increased the expression of iNOS, the enzymes were not totally activated and the increase in NOS activities (~1.5 folds) were not as much as the increase in NO production (~4.2 folds). Therefore, the high level of NO may be attributed to, on the one hand, the enhancement of intracellular NOS activities; on the other hand, the plasma may act as a NO donor through the free radicals reaching and penetrating into the cells, forming nitrite with high stability to maintain the long-term high RNS levels.

### APRTP-Js Treatment Compromised Cellular Antioxidant Defense System in HepG2 Cells

**Figure 5:**
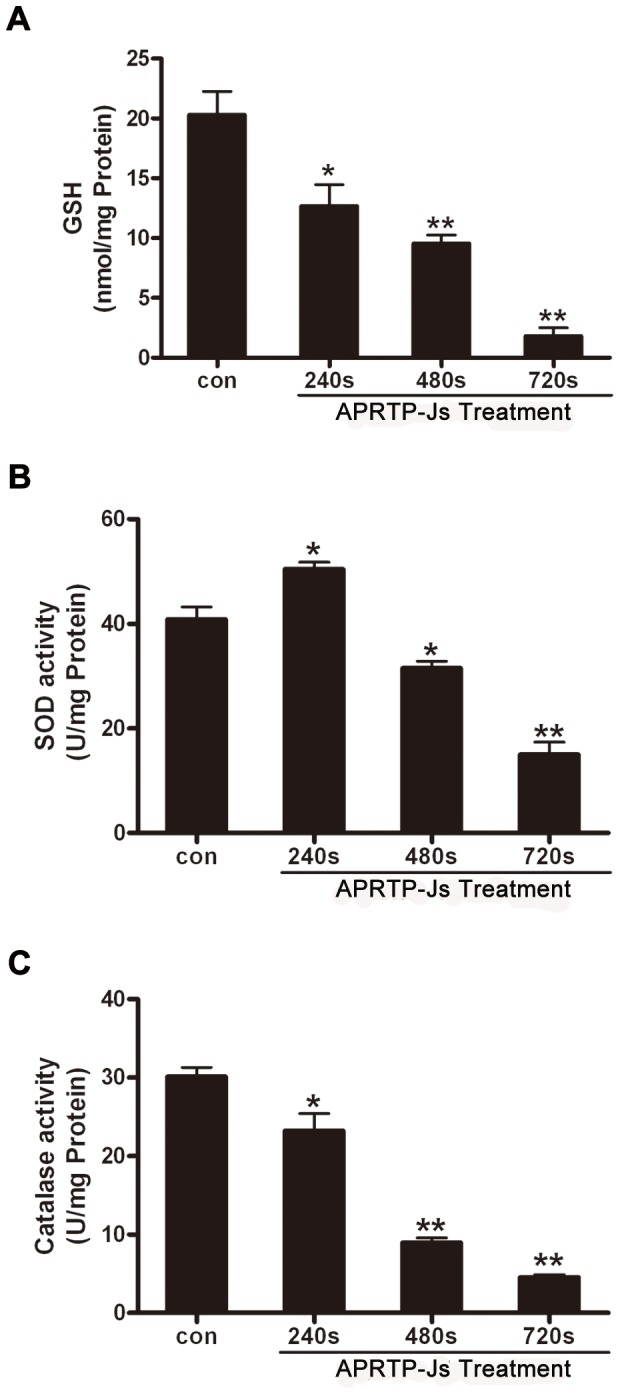
APRTP-Js Treatment compromised cellular antioxidant defense system in HepG2 cells. Cells were treated without or with APRTP-Js for 240 s, 480 s and 720 s, respectively. 24 h later, the cells were harvested and lysed using cell lysis buffer. The samples were centrifuged and the supernatants were used for antioxidant activities assay. (A) The effect of APRTP-Js treatment on total GSH content in HepG2 cells. (B) The effect of APRTP-Js treatment on the SOD activity in HepG2 cells. (C) The effect of APRTP-Js treatment on the catalase activity in HepG2 cells. Values are means ± SEM obtained from three independent experiments. The asterisk represents statistical significance in comparison with control value (*p < 0.05 and **p < 0.01).

The accumulation of ROS and RNS indicated the oxidative and nitrative stress of cells and an appropriate balance between oxidation and anti-oxidation of the cells was important for cellular resistance to oxidative/nitrative stress. To evaluate the case, we measured the capacity of cellular antioxidant defense system. SOD and catalase enzymatic activities, as well as GSH content, were shown in Figure 5.

GSH plays an important role in scavenging free radicals by its conversion to GSSG and is a useful index for determining the capacity of cellular antioxidant defense system [41,42]. Spectrophotometric analysis revealed a marked decrease in total GSH content in cells with plasma exposure (Figure 5A). Our data showed ~40% (p<0.05), ~50% (p<0.01) and ~87% (p<0.01) declines in GSH content in HepG2 cells with APRTP-Js treatment for 240 s, 480 s and 720 s, respectively, compared to normal control. This observation suggested that HepG2 cells with APRTP-Js treatment were poorly equipped to present a glutathione antioxidant defense system.

SOD, which catalyzes the dismutation of the O_2_^·-^ into H_2_O_2_ and oxygen, is one of the most important antioxidant enzymes and plays an important role in the balance between oxidation and anti-oxidation of the cells. Figure 5B was the analysis result of total SOD activity. Interestingly, we observed a ~25% increase in SOD activity in cells with low-dose plasma treatment (240 s), compared to control. However, the SOD activity declined to ~75% (p<0.05) and ~37% (p<0.01) of control when cells suffered with high-dose of plasma treatment (480 s and 720 s), respectively. There was a possibility that: short-time APRTP-Js treatment (240 s) made the cells to be subjected to oxidative stress and intracellular SOD activity was increased to scavenge the increased free radical to avoid oxidative damage of cells. However, when the reactive oxygen species were generated too much and exceeded the scavenging capacity of the antioxidant, the cellular antioxidant defense system was compromised with decreased SOD activity (cells with APRTP-Js treatment for 480 s or 720 s).

Moreover, the catalase activity in groups with 240 s, 480 s and 720 s of APRTP-Js treatment were reduced to ~80% (p<0.05), ~30% (p<0.01) and ~16.7% (p<0.01) of control, respectively (Figure 5C). The catalase activity provided antioxidative capability against oxidative stress by converting toxic H_2_O_2_ to non-toxic H_2_O and oxygen, thus, the decrease of catalase activity implied the compromised antioxidant system in HepG2 cells with plasma exposure.

Altogether, our data showed a marked decline in the cellular antioxidant capacity and the balance between oxidation and anti-oxidation of the cells was destroyed.

### APRTP-Js Treatment Caused Oxidative and Nitrative Damage in HepG2 Cells

**Figure 6:**
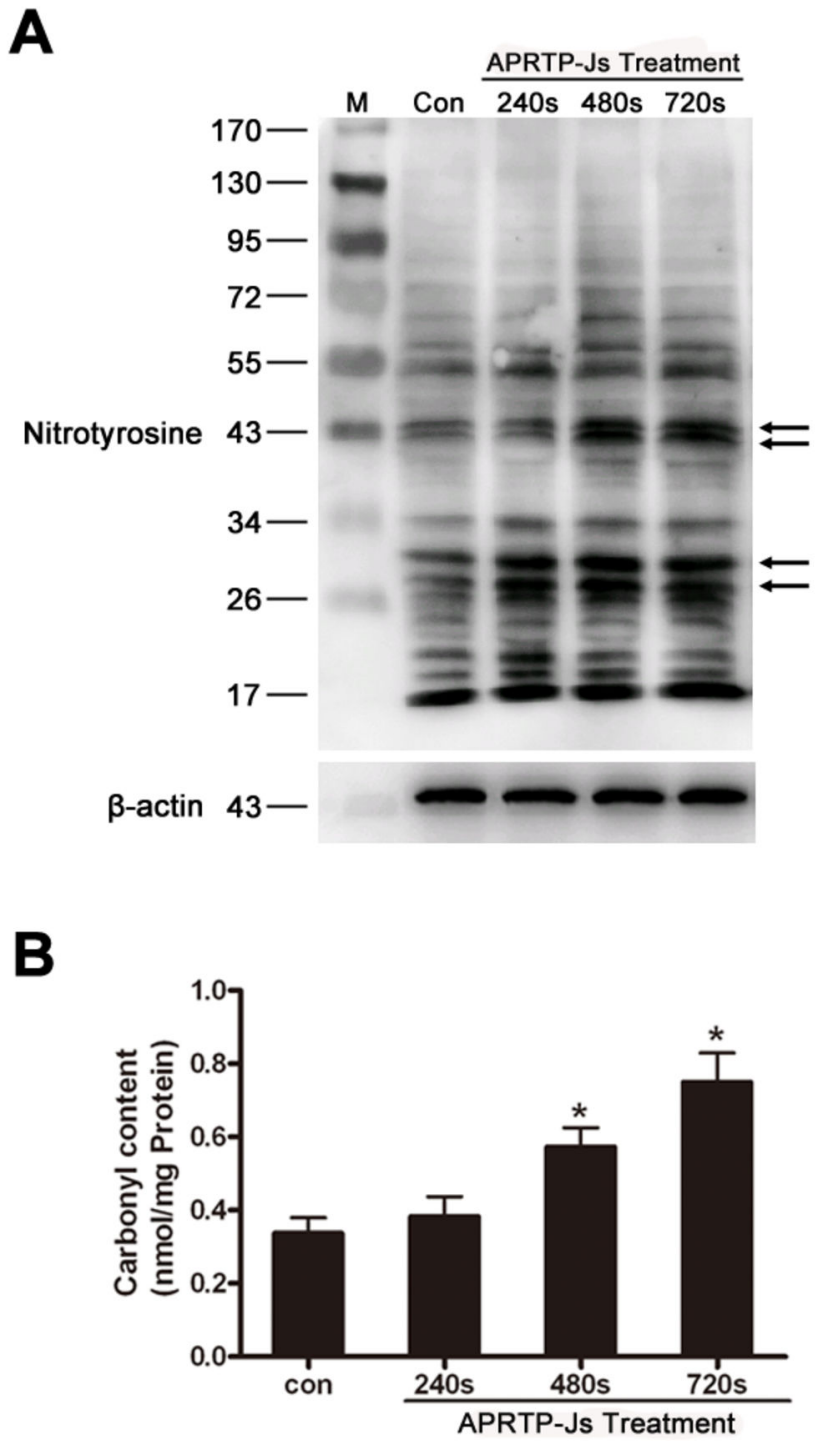
APRTP-Js treatment induced oxidative and nitrative damage in HepG2 cells. (A) Status of total protein nitration. The total nitration statuses of proteins were studied using a nitrotyrosine antibody by Western blot in cells with or without APRTP-Js treatment. The deepened nitrotyrosine epitopes were indicated by arrows. (B) Status of protein carbonyl content. The protein carbonyl content was measured using a commercial assay kit. Values are means ± SEM obtained from three independent experiments. The asterisk represents statistical significance in comparison with control (*p < 0.05 and **p < 0.01).

Proteins are likely to be a major target for oxidative and nitrative damage due to their abundance in cells. Because RNS exert their biological activities mainly through nitration of multiple target proteins with tyrosine residues, we also determined tyrosine nitration of cells using Western blot. The level of nitrated proteins, although normally low, greatly increased in plasma-treated HepG2 cells. The band intensity of those original nitrotyrosine epitopes deepened, especially those proteins with molecular masses of approximately 26~30 and 42~44 kDa (Figure 6A). These results demonstrated that exposure of HepG2 cells to plasma could lead to a dose-dependent increase in tyrosine nitration of cellular proteins.

Carbonyl groups are introduced to proteins as a result of oxidative cleavage of the peptide backbone. The formation of carbonyls thus serves as an important indicator of oxidative protein damage. The next step of the present study was to assess the effect of plasma on protein oxidative damage by measuring carbonyl formation in the cells. Our results showed that, compared to control, the levels of protein carbonyl groups were significantly increased in APRTP-Js-treated groups, especially in groups exposed to plasma for 720 s (p<0.01) (Figure 6B). It is generally accepted that carbonyl formation can lead to structural alterations and loss of biological function of proteins [43]. The result suggested that the cells under APRTP-Js treatment suffered intracellular oxidative damage which could further aggravate cell injuries.

### APRTP-Js Treatment Disturbed Intracellular Calcium Homeostasis of HepG2 Cells

**Figure 7:**
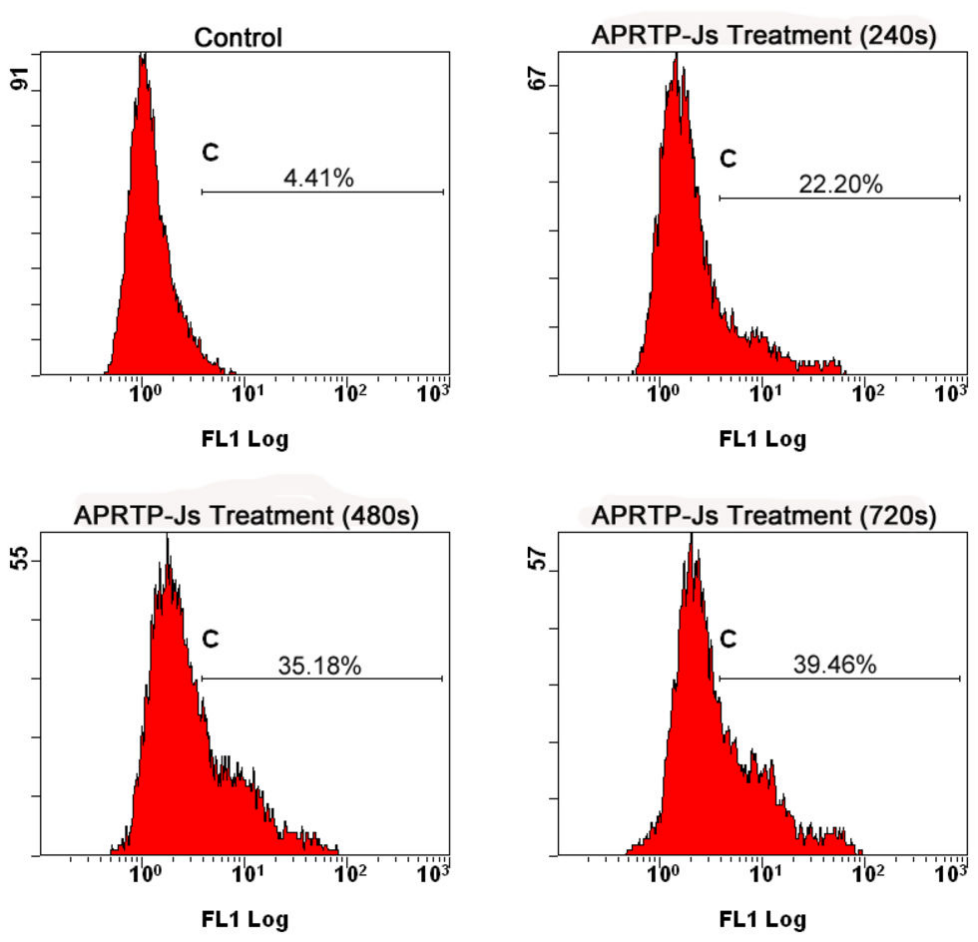
APRTP-Js treatment disturbed intracellular calcium homeostasis. Cells were treated without or with APRTP-Js for 240 s, 480 s and 720 s, respectively. After continuous culturing for 24 h, the cells were loaded with calcium probe Fluo-3/AM and the intracellular free Ca^2+^ was measured using a FACScan flow cytometer.

Calcium homeostasis is essential for many cellular functions, such as protein folding, processing, transport and signal transduction. Also, calcium can induce apoptosis in response to a variety of stimuli [44]. To determine whether plasma exposure affected the calcium homeostasis of HepG2 cells, the Fura-3/AM fluorescence method was used. As shown in Figure 7, percentage of cells stained by Fura-3/AM increased from 4.41% to 22.2%, 35.18% and 39.46% when the APRTP-Js treatment time varied from 0 to 240 s, 480 s and 720 s, respectively. The result demonstrated that there was an accumulation of the intracellular free Ca^2+^ and the calcium homeostasis was disturbed in HepG2 cells with APRTP-Js treatment.

### APRTP-Js Treatment Triggered ER Stress in HepG2 Cells

**Figure 8:**
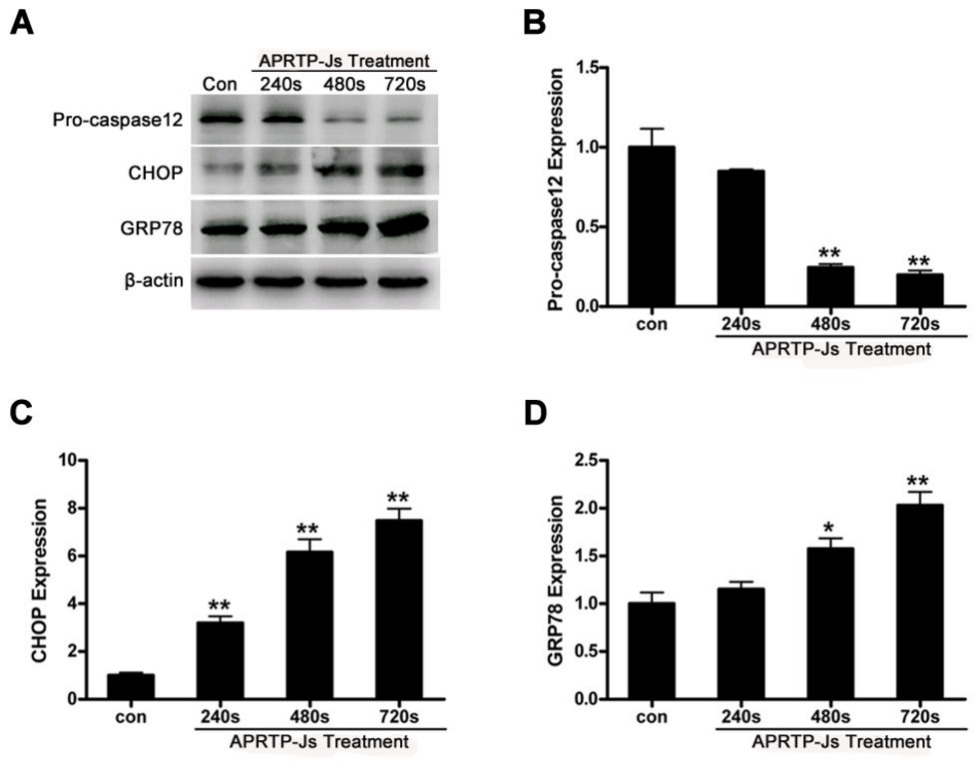
APRTP-Js treatment triggered ER stress in HepG2 cells. (A) Representative autoradiographs of pro-caspase12, GRP78, and CHOP. Detection of β-actin was carried out to confirm the equal loading of proteins. (B–D) The corresponding densitometric analysis of pro-caspase12, GRP78, and CHOP, respectively, and data were normalized using the β-actin signal. The asterisk represents statistical significance in comparison with control (*p < 0.05 and **p < 0.01).

The pro-apoptotic effects of Ca^2+^ are mediated by a diverse range of Ca^2+^ sensitive factors that are compartmentalized in various intracellular organelles including the ER. Perturbation of calcium homeostasis is expected to induce ER stress. ER stress is an adaptive mechanism triggered to allow cells survival upon challenges they are subjected to. However, if the challenges are not alleviated, these same signaling pathways are responsible for a pro-death program aiming at clearing up cells unable to survive. The downstream signaling of ER stress is mainly transduced through GRP78 and CHOP, which are known to promote ER stress-induced apoptosis [36,45]. Caspase12, the executioner of the ER stress-mediated apoptosis, is specifically localized to the ER and has been reported to be cleaved during ER stress-induced apoptosis [38]. The effects of plasma treatment on the expressions of GRP78, CHOP, and pro-caspase12 are shown in Figure 8A. The corresponding densitometric analysis of each band was quantified of pro-caspase12 (Figure 8B), CHOP (Figure 8C), and GRP78 (Figure 8D). After exposure to plasma for 240 s, 480 s, 720 s, the relative expressions of GRP78 and CHOP were increased significantly with dose-dependent manner, compared to control. Consistent with these, caspase12 was activated after APRTP-Js treatment, which possessed a significant decrease in its precursor (Figure 8B). These changes of protein expressions in the APRTP-Js treated cells indicated the occurrence of ER stress.

### NAC Attenuated the Apoptosis Induced by APRTP-Js Treatment

**Figure 9:**
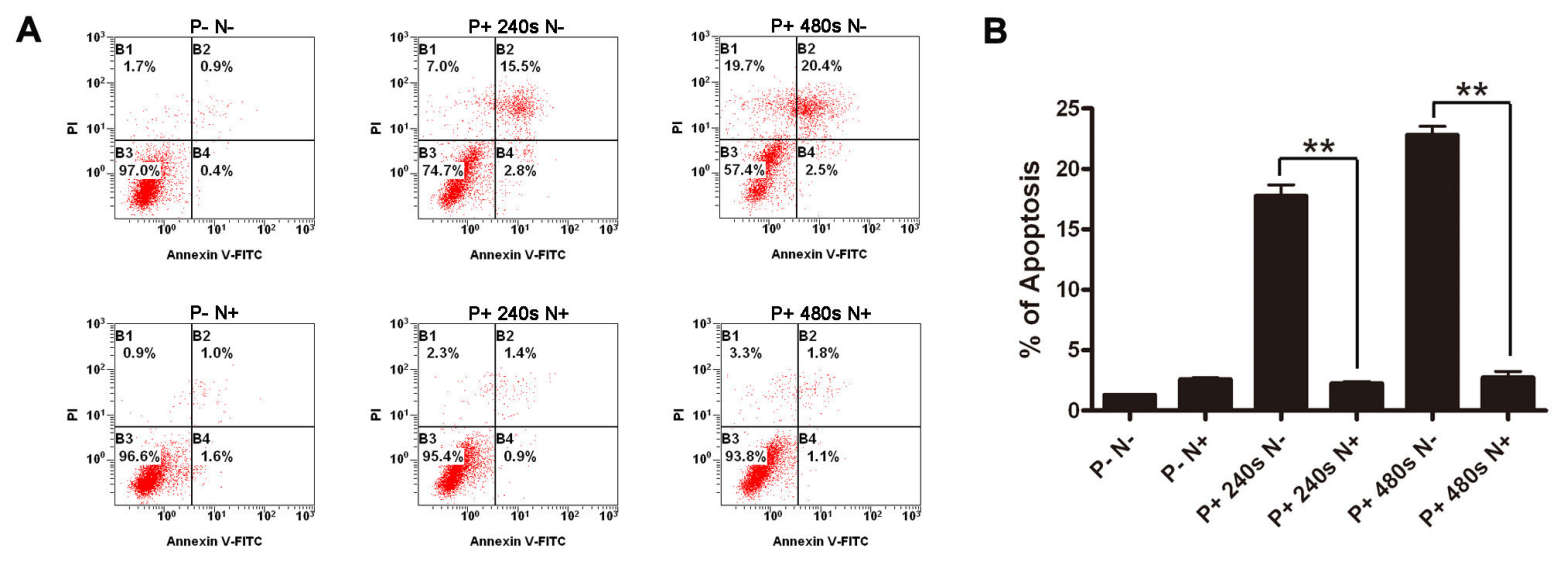
NAC attenuated the apoptosis induced by APRTP-Js treatment. (A) HepG2 cells were incubated for 1h with 10 mM NAC (N+) or cell culture medium (N-), followed by treatment with the indicated dose of plasma. Apoptosis was measured 24 h after APRTP-Js treatment by collecting and staining the cells with annexin V-FITC/PI. Samples were run on the flow cytometry. P- N-: control group without any treatment; P+ 240s N-: cells with 240 s plasma exposure only; P+ 480s N-: cells with 480 s plasma exposure only; P-N+: cells with NAC pre-treatment only; P+ 240s N+: cells with NAC pre-treatment followed by 240 s plasma exposure; P+ 480s N+: cells with NAC pre-treatment followed by 480 s plasma exposure. (B) Percentage of apoptotic cells was determined from flow cytometry results.

To obtain direct evidence that supports a causative link between overproduction of ROS/RNS and apoptosis of HepG2 cells, a comparative study using antioxidant was conducted. Prior to APRTP-Js treatment, HepG2 cells were pre-treated with or without free radical scavenger NAC. Plasma treatment was carried out as mentioned above. After continuously cultured for 24 h, HepG2 cells were harvested and apoptotic activity was assayed using annexin V-FITC/PI staining. Cells without any treatment (P-N-) and cells with NAC pre-treatment only (P-N+) were used as controls. We found that NAC pretreatment almost completely attenuated apoptosis [(P+ 240s N+) group and (P+ 480s N+) group], compared correspondingly to cells with the same plasma treatment-dose in the absence of NAC [(P+ 240s N-) group and (P+ 480s N-) group] (Figure 9). APRTP-Js treatment with dose of 240 s and 480 s induced apoptosis of ~18.3% and 22.9% of the total cells, respectively, which was reduced to ~2.3% and 2.9%, respectively, in cells with NAC pre-treatment. NAC itself had almost no effect on HepG2 cells [(P-N+) group]. These results strongly suggested that overproduction of ROS/RNS was the primary mechanism that mediated the apoptosis induced by plasma in HepG2 cells.

## Discussion

Recently, APRTP-Js have found entry into medicine where they are potentially applied in blood coagulation, tissue sterilization, cancer therapy, root canal treatment and diverse other applications. Induction of apoptosis is an important event in cells in response to APRTP-Js treatment. It is generally accepted that ROS and RNS are the main components of plasma. However, how these emitted species mediate the biological pro-apoptotic effect of plasma remains elusive. In this study, we demonstrated the induction of apoptosis in a human hepatoma cell line (HepG2) *in vitro* by exposure to APRTP-Js and presented some aspects of the underlying biological mechanisms.

Previously, other groups have shown that plasma was able to initiate apoptosis in diverse cell lines, including Hela cancer cells [46], melanoma skin cancer cells [47], colorectal cancer cells [48] and liver cancer cells [49,50]. To confirm the pro-apoptotic effect of plasma, annexin V-FITC/PI and Hoechst 33342 staining were utilized in HepG2 cells after APRTP-Js treatment. APRTP-Js were shown to induce apoptosis in HepG2 cells at a dose of 240 s exposure or higher (Figure 3A). During apoptosis, cells underwent a series of complex morphological changes, including nuclear condensation and degradation of DNA into nucleosomal fragments (Figure 3B). In our conditions, plasma is generated in ambient air and the main active species are OH^·^, N_2_^+^, He, O, as well as the NO and O_2_^·-^ generated subsequently. It is generally accepted that the generation or addition of reactive species can cause cell death by apoptosis. To identify the components involved in plasma-induced apoptosis, the content of ROS and RNS were measured in HepG2 cells 24 h after APRTP-Js treatment (Figure 4). Remarked increase in ROS and RNS content was found, indicating the accumulation of ROS and RNS in the apoptotic process. The use of NAC provided direct *in vitro* evidence to support that overproduction of ROS/RNS played a causative role in plasma-induced apoptosis of HepG2 cells (Figure 9).

ROS and RNS are particularly short-lived species owing to their high chemical reactivity. However, we found a long-term (24 h) production of ROS and RNS (Figure 4 A and B). Therefore, we assumed that plasma exposure led to the physiological and biochemical changes to produce the ROS/RNS within the cells. ROS produced extracellularly by APRTP-Js may immediately move across the cell membrane by active transport across the membrane, transient opening of pores in the membrane, then interact with amino acids and proteins and finally lead to production of long-lived reactive hydroperoxides or activation of pathways that modify intracellular ROS concentration. Meanwhile, generation of ROS is an inevitable outcome of oxygen-dependent respiration within the cells especially suffering damage. These in turn may aggravate intracellular injuries and activate signaling pathways leading to apoptosis. NO can be synthesized endogenously by a family of NO synthase (cNOS and iNOS). The cNOS are Ca^2+^-dependent and constitutively expressed in a wide variety of cells. While, iNOS is Ca^2+^ independent and is induced to express in cells of immune system and other cells in response to various stimuli. Thus, the NO production can be regulated on demand. We found that APRTP-Js treatment could cause increased enzyme activities and expression of iNOS, especially in group of 720 s APRTP-Js treatment. Interestingly, the increase in NOS activities (~1.5 folds) was not as much as the increase of NO (~4.2 folds) (Figure 4B, C, D and E). The excess NO production may be due to the diffusion of NO (emitted by APRTP-Js) into the cells to form stable nitrite that can be detected.

Although, there is an accumulation of ROS and RNS that are cytotoxic, the cells are equipped with an extensive antioxidant defense system to combat ROS/RNS, either directly by interception or indirectly through reversal of oxidative/nitrative damage. The defense system composes of the nonenzymatic antioxidants such as GSH and enzymatic antioxidants such as SOD, catalase, GPx, GR and so on. Under normal conditions, the antioxidant system minimizes the harmful effects by intercepting reactive species before they can damage intracellular targets. However, when free radicals overcome the defense system of the cell and redox homeostasis is altered, the result is oxidative/nitrative stress. In our study, the defense system of cells treated with APRTP-Js was compromised, as evidence by the limited activities of GSH, SOD and catalase (Figure 5). These indicated that the cells were subject to oxidative and/or nitrative stress after APRTP-Js treatment.

Furthermore, persistent exposure of cells to oxidative and/or nitrative stress can lead to increases in the cellular levels of oxidatively/nitratively modified proteins and eventually to impaired function. In order to verify oxidative and/or nitrative injuries involved in plasma-induced apoptosis in HepG2 cells, we monitored the formation of carbonyl and nitrotyrosine in cells after APRTP-Js treatment for 24 h. To our knowledge, this is the first time to put forward the roles of tyrosine nitration and carbonylation involved in the plasma-induced apoptosis.

Protein modifications of carbonyl formation and nitrotyrosine can affect various biological effects such as enzymatic activities, cell signaling and cytoskeletal structure which could ultimately lead to cell apoptosis. Protein carbonylation is usually being recognized as an indicator of oxidative stress as it arises in a high level and is very easy to detect, relative to other oxidative modifications [51]. In our experiment, a significant increase of carbonyl content was observed in groups with plasma exposure, compared to control (Figure 6B). Since carbonylation is non-repairable and involves the introduction of a relatively large and reactive group onto the protein, it can cause deleterious effects on protein’s properties, involving its structure, intermolecular cross-linking, activities and degradation [52–54]. Consequently, cells that have large numbers of carbonylated proteins may be reduced to dysfunction.

Moreover, using Western blot, we evaluated the level of nitrated proteins and found that those original nitrotyrosine aggravated in APRTP-Js -treated HepG2 cells. Interestingly, unlike the common way of nitrification (proteins of high-molecular-weight were more sensitive to be nitrated due to the relatively more tyrosine residues at the surface of the proteins, determining the high susceptibility to be nitrated, compared to low-molecular-weight proteins), the low-molecular-weight proteins were more likely to be nitrated in our experiments, especially proteins with molecular masses of approximately 26~30 and 42~44 kDa (Figure 6A). This looks like a selectively nitrative modification of proteins, maybe some enzymes and cytoskeletal proteins, of which nitration could cause protein inactivation and cell dysfunction. Accumulation of damaging modifications may lead to a positive feedback loop and eventually to irreversible alterations that cause cell apoptosis.

Overproduction of free radicals and redox modifications may lead to inactivation of functional enzymes and cytoskeletal proteins as suggested in the case of SOD and actin by tyrosine nitration [55,56]. The SOD family is composed of two important intercellular metalloenzymes (Cu/Zn–SOD and MnSOD), whose molecular weight are 26~32 kDa. Actin, a ~42 kDa protein which constitutes 5% or more of cell proteins, is susceptible to be nitrated. Schopfer et al. revealed that nitrative modification of cytoskeletal proteins could activate apoptotic signaling pathways of cells [57]. The nitration of actin can cause a disorganization of the actin fibers, alteration of cytoskeleton dynamics that lead to ER stress and cell death. Figure 6A showed substantial increase in the tyrosine nitration of proteins with molecular weight of approximately 26~30 and 42~44 kDa. We have therefore proposed that the SOD and actin were some of the target proteins of nitrative modification in APRTP-Js-treated HepG2 cells. More experiments are needed to confirm this assumption.

Oxidative/nitrative stress and ER stress are closely linked events [58,59]. Since the relatively limited antioxidant capacity and physiological conditions within the ER, ER proteins may be particularly sensitive to protein oxidation/nitration [60]. Meanwhile, perturbance of cellular redox state or accumulation of reactive species could directly and/or indirectly affect ER homeostasis, which will result in deleterious consequences [61,62]. The ER is essential for calcium storage. In case of ER stress, intracellular calcium releases, causing the disturbance of calcium homeostasis in cells. As shown in Figure 7, we found that calcium homeostasis was disturbed in HepG2 cells with plasma exposure, which was indicative of ER stress. Simultaneously, the expressions of GRP78 and CHOP were up-regulated, combined with activation of caspase12, which confirmed the occurrence of ER stress (Figure 8). GRP78 is a key regulator in ER stress signaling that has a dynamic capacity to regulate the balance between cell survival and apoptosis in ER-stressed cells. In the early stage of ER stress, the GRP78 is activated to alleviate the stress, thereby restoring the ER homeostasis. However, if the perturbance cannot be alleviated, the downstream pro-apoptotic proteins of CHOP and caspase12 are induced, mediating the process of ER stress-induced apoptosis.

Altogether, we provide insight into the plasma-induced apoptosis *in vitro* from the point of view in oxidative and nitrative stress, and at the same time, the occurrence of ER stress. The plasma not only functions as a donor of active species to attack the cellular targets; but also, activates the cellular pathways that modify the intracellular production of ROS and RNS. These different species (ROS and RNS) may act synergistically and provide a basis for stress-associated alterations that culminate into apoptosis in HepG2 cells. Considering the concomitant occurrence of cellular oxidative and nitrative stress, alteration of intracellular calcium homeostasis, ER stress and apoptosis, the problem of the mutual relationships among these events seems to be of particular relevance. ER stress and alterations in redox status may interact in a global way to mediate the pro-apoptotic effect of plasma. The better understanding of this process may provide us new insights into the evaluation of plasma medicine with the safe utilization of plasma technology.

Most importantly, due to the high reactive efficiency, APRTP-Js are proved to be a more effective approach to induce apoptosis, compared to traditional means. Meanwhile, APRTP-Js can be attached to the end of a probe, thereby, they have the potential benefit of being able to target specific areas of the body that are technically difficult to reach such as internal organs with precise control of treatment area and depth. Since the reactive species emitted by plasma jets are ephemeral, the application of APRTP-Js may be of no hazardous residual. Moreover, APRTP-Js can induce cell death through apoptosis other than necrosis, thus, systemic side effects can be potentially avoid. However, ROS/RNS at low concentrations has been reported to promote cell proliferation [17]. For the treatment of cancer, the amount of reactive species generated by APRTP-Js can be controlled by regulating the parameters and working gases, allowing for the fine-tuning of the therapeutic effect, from stimulating cell proliferation to inducing apoptosis. Based on the apoptotic effect on HepG2 cells, APRTP-Js are expected to become a novel means for clinical cancer therapy. Further investigations are needed to ascertain the *in vivo* anti-tumor effect of the APRTP-Js.
